# Selection and Optimization of High-Yielding DNA Isolation Protocol for Quantitative Analyses of Methanogenic Archaea

**DOI:** 10.3390/microorganisms10030523

**Published:** 2022-02-28

**Authors:** Agata Anna Cisek, Iwona Bąk, Ilona Stefańska, Marian Binek

**Affiliations:** Department of Preclinical Sciences, Institute of Veterinary Medicine, Warsaw University of Life Sciences, Ciszewskiego 8, 02-786 Warsaw, Poland; dabrowskaiwona88@gmail.com (I.B.); ilona_stefanska@sggw.edu.pl (I.S.); marian_binek@sggw.edu.pl (M.B.)

**Keywords:** archaea, bead-beating, DNA isolation, gut microbiota, *mcrA* gene, methanogens, real-time PCR, sonication

## Abstract

Methanogenic archaea are a functionally important component of the intestinal microbiota of humans and animals, participating in the utilization of detrimental hydrogen produced during gut fermentation. Despite this, archaeal DNA has rarely been found in intestinal microbiome analyses, which prompts the need to optimize detecting procedures of these microorganisms, including the DNA isolation step. Three commercially available kits for DNA isolation and one extra purification kit that removes PCR inhibitors were evaluated on chicken droppings. In addition, different variants of mechanical lysis and a double elution were tested to ensure the maximum efficiency of DNA isolation from archaea as well as bacteria. A quantitative real-time PCR was used to monitor the optimization progress. As a result, the combination of the selected Genomic Mini AX Bacteria+ kit with a 2-min-long sonication by ultrasonic probe and enzymatic pretreatment gave excellent extraction efficiency rates for DNA of methanogenic archaea (an approximate 50-fold increase compared to the standard enzymatic lysis described by the producer) and, at the same time, provided optimal protection of DNA extracted from bacteria susceptible to enzymatic lysis. The presented results indicate that the optimized protocol allows for highly efficient extraction of total DNA, which is well-suited for quantitative microbial analyses by real-time PCR.

## 1. Introduction

The gut microbiome has recently been gaining more and more attention due to the increasing awareness of the microbiota’s role in maintaining the host’s health and well-being [1]. A difference between health and illness often relies on a quantitative microbial imbalance. For this reason, quantitative studies of microbiota require proper isolation of total DNA from stool or intestinal samples. It stands to reason, then, that DNA isolation protocols should be designed to include as many microorganisms as possible; therefore, not only bacteria but archaea and single-celled eukaryotes as well. Out of these three, archaea seem to be the most difficult to extract DNA from.

The archaeal cell wall composition is diverse, but, if only focusing on methanogens that colonize guts of vertebrates, it is mostly built by an S-layer (*Methanococcus*), an S-layer with a protein sheath (*Methanospirillum*), an S-layer with methanochondroitin (*Methanosarcina*), pseudomurein with an S-layer (*Methanopyrus*), or pseudomurein with heteropolysaccharide and glutaminylglycan (*Methanosphaera*, *Methanobacterium*, and *Methanobrevibacter*) [2]. It is safe to say that pseudomurein is the most common archaeal cell wall component, as it builds cell walls of *Methanobrevibacter*, the most predominant archaeal genus in chicken ceca, and presumably in other parts of the intestines as well [3]. Pseudomurein is similar to murein found in bacteria. Murein undergoes degradation by lysozyme at the 1,4-glycosidic bond site between N-acetylglucosamine (NAG) and N-acetylmuramic (NAM). Pseudomurein however, is built by NAG connected to N-acetyltalosaminuronic acid (NAT) and N-acetylgalactosamine by β-1,3-glycosidic bonds [4], and this peculiar sugar linkage makes archaea resistant to lysozyme. Therefore, proteinase K is the only routinely used enzyme that can be successful in the extraction of archaeal DNA. For this reason, it is necessary to search for a more complex and optimized DNA extraction method (rather than just simple standard procedures applied to bacteria).

The aim of this study was (1) to develop a low-priced, universal DNA isolation method that would result in a very effective extraction of DNA from the archaea present in stool-like samples (and intestinal contents as well) but at the same time would not lead to a drastic damage of DNA isolated from other microbes that are less resistant to lysis—like Gram-negative bacteria—and (2) to design a DNA isolation method which would be PCR-compatible. 

**Figure 1:**
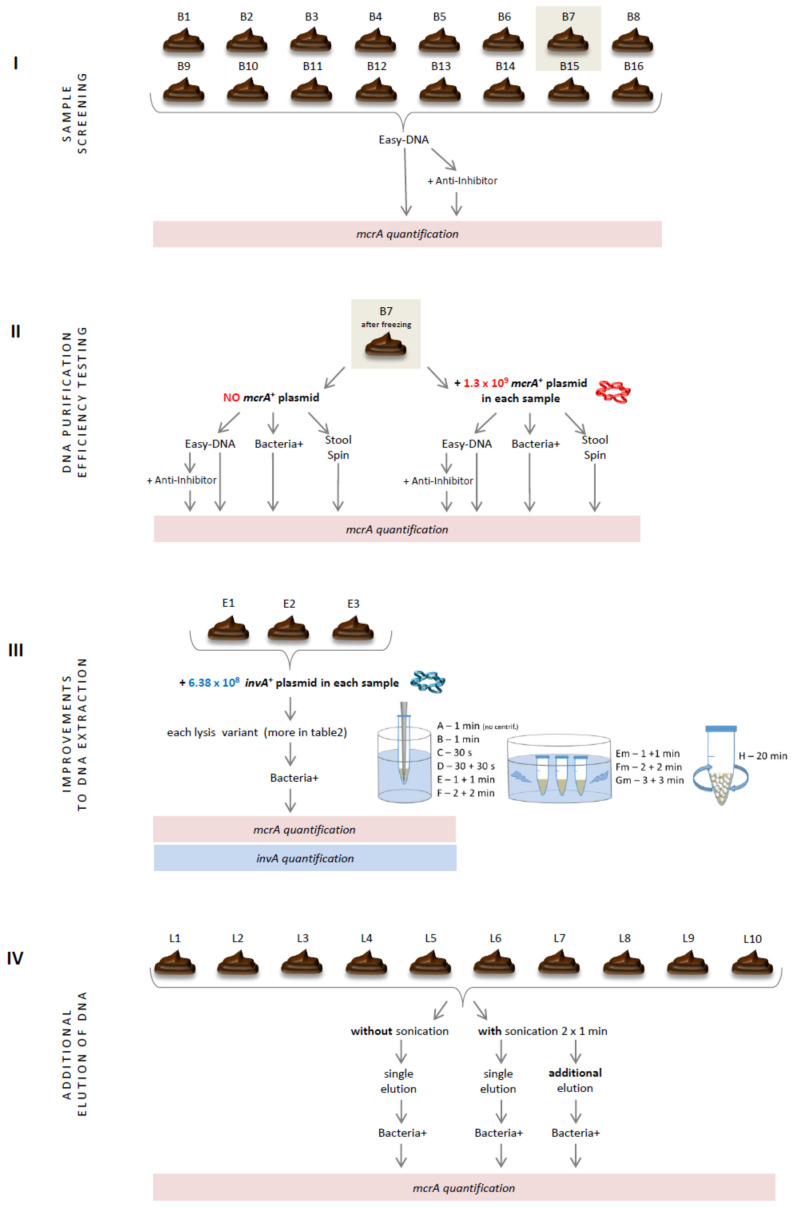
Schematic representation of research.

This study comprised four experiments performed on chicken dropping samples (Figure 1). In the first one, samples were screened for the presence of methanogenic archaea with the use of real-time PCR and DNA templates isolated with the commercially available DNA isolation kit that seemed to be one of the most efficient and best-working on avian intestinal samples [5]. Faced with unfavorable results, two other DNA isolation kits varying in the purification strategy were tested (Section 2.2). After these initial experiments, one kit was selected and analyzed in detail. Finally, several improvements to the producer’s isolation protocol of the selected kit were tested, including additional mechanical lysis (Section 2.3) and double elution of DNA from the purification columns (Section 2.4). The results of these four experiments are presented in numerical values for better understanding of the relevance of each improvement incorporated into the DNA isolation protocol.

## 2. Materials and Methods

### 2.1. Sample Screening

16 samples of fresh droppings, from B1 to B16, were collected from the floor of a henhouse of free-range chickens. Of each sample, 100 mg was weighed into sterile microtubes and subjected to DNA isolation, and a dropping sample residue was portioned and frozen for further analysis. DNA extraction was performed with the Easy-DNA kit (Invitrogen, Thermo Fisher Scientific, Waltham, MA, USA) according to the manufacturer’s instructions from protocol #3—Small Amounts of Cells, Tissues, or Plant Leaves including the suspension of samples in 200 µL PBS. In the end, one-half of the DNA (50 µL) was additionally purified with the Anti-Inhibitor kit (A&A Biotechnology, Gdynia, Poland). The concentration and purity of DNA was measured with Nanodrop 1000 (Thermo Fisher Scientific, Waltham, MA, USA).

All DNA samples were used in quantitative real-time PCR (qPCR) with primers targeting the fragment of *mcrA* gene encoding the alpha subunit of methyl-coenzyme M reductase in methanogenic archaea (Table 1). The reaction mixture included 15 µL RT HS-PCR Mix Probe (A&A Biotechnology), 0.75 µM of primers mcrA_F3 and mcrA_R1, 0.25 µM of mcrA_probe2, 3 µL of DNA, and water to reach a final volume of 30 µL. The reactions were performed in a LightCycler 480 (Roche, Basel, Switzerland) as follows: initial denaturation at 95 °C for 3 min, and 45 cycles comprising denaturation at 95 °C for 20 s, and annealing/extension/fluorescence acquisition at 60 °C for 40 s. Absolute quantification was done based on a standard curve generated for serial dilutions of linearized plasmid construct mcrA_MB containing a 472-bp long *mcrA* gene fragment from *Methanobrevibacter* sp. D5 (GenBank acc. no. KF214818.1:976-1447). Linearization was performed with the use of a single cutting VspI restriction enzyme (Thermo Fisher Scientific, Waltham, MA, USA). Gel electrophoresis was done in order to select only the linearized form of *mcrA*-positive plasmid, which was then purified with the Basic DNA Purification Kit (EURx). A number of copies in standards was calculated from the known concentration of DNA (measured with the Quantus fluorometer and QuantiFluor dsDNA System (Promega Corporation, Madison, WI, USA)) and the length of the plasmid by using the Science Primer tool [6]. The quantification results were calculated into the number of methanogenic archaeal cells in 1 g of a dropping sample.

### 2.2. DNA Purification Efficiency Testing

The B7 dropping sample, which previously tested negative for the presence of methanogenic archaea, was selected for further analysis. Portions of only 55 mg were weighed to avoid a potential negative effect of column overload, then the samples were thawed in their intended lysis buffers. Half of the tubes were enriched with 1.3 × 10^9^ *mcrA*-positive plasmid as an internal control of DNA purification efficiency (IC). The number of copies per µL was determined as described previously. The Easy-DNA kit—with and without an additional anti-inhibition purification step—and two other DNA isolation kits, i.e., Genomic Mini AX Bacteria + kit (A&A Biotechnology), and Genomic Mini AX Stool Spin kit (A&A Biotechnology) were used. The experiment was performed in triplicate.

Isolation of total DNA with the use of the Easy-DNA kit was performed as described previously. As for the GMA Bacteria+ and GMA Stool Spin kits, dropping samples were suspended in 1.5 mL and 900 µL LS buffer, respectively. A total of 20 µL of proteinase K (20 mg/mL), and 4 or 3 µL of lysozyme (10 mg/mL) was added depending on a kit. Samples were incubated at 50 °C for 30 min. Then 2 µL RNase A (10 mg/mL) was added, and samples were incubated at room temperature for 5 min. Next, isolation steps were performed according to the manufacturer’s instructions. The concentration and purity of DNA samples were measured with Nanodrop 1000 (Thermo Fisher Scientific, Waltham, MA, USA).

A *mcrA* qPCR was performed on LightCycler 480 with the previously described reaction mixture and thermal parameters; however, the quantification results were calculated into no. of *mcrA* copies in a total volume of extracted and purified DNA. A *t*-test for independent samples was used to determine statistically significant differences between DNA isolation methods. Statistical analyses were performed in TIBCO Statistica 13.3 (TIBCO Software Inc., Palo Alto, CA, USA) and Microsoft Office Excel 2016.

### 2.3. Improvements to DNA Extraction

**Figure 2:**
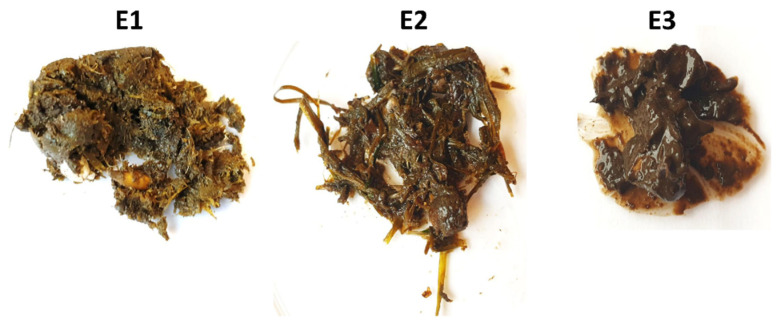
The dropping samples E1–E3 varying in texture and content of plant debris: E1—some plant debris, E2—mostly grass, E3—most likely cecal droppings, no plant debris.

Three fresh samples of droppings, i.e., E1, E2 and E3, were collected from the same country house chicken coop. Dropping samples varying in the texture and content of plant debris (Figure 2) were divided into 200 mg portions and underwent lysis by different variants as shown in Table 2. Large plant particles were removed by as far as possible. In order to ensure the best reaction conditions for enzymes, samples were suspended in 500 µL BS buffer (A&A Biotechnology), 30 µL lysozyme (10 mg/mL), and 7 µL mutanolysin (10 U/µL). Suspensions were incubated at 37 °C for 10 min, and then at 50 °C for 30 min. In the next step, proteinase K was added in a volume of 35 µL (20 mg/mL) along with 500 µL LS lysis buffer, and the mixture was further incubated at 50 °C for 60 min.

Exactly 6.38 × 10^8^ copies of the pJET1.2 plasmid carrying the 200 bp-long fragment of invasion gene *invA* from *Salmonella enterica* subsp. *enterica* serovar Typhimurium ATCC 14028 (acc. no. CP034230.1:998383-998582) was added to each tube 30 min prior the end of incubation as an internal control of mechanical lysis (IC2). Negative controls, i.e., dropping samples E1–E3 without the plasmid, were also included to confirm the absence of *Salmonella* in the original samples. The *invA* gene was chosen as a target since it is specific to *Salmonella*, and oligonucleotides designed to detect *invA* were shown to be highly sensitive, specific, and excellent for quantification purposes. Moreover, by choosing *Salmonella* as a target, we would be able to establish its presence by yet another method—cultivation—if for some reason our samples had tested positive for the *invA* gene.

**Figure 3:**
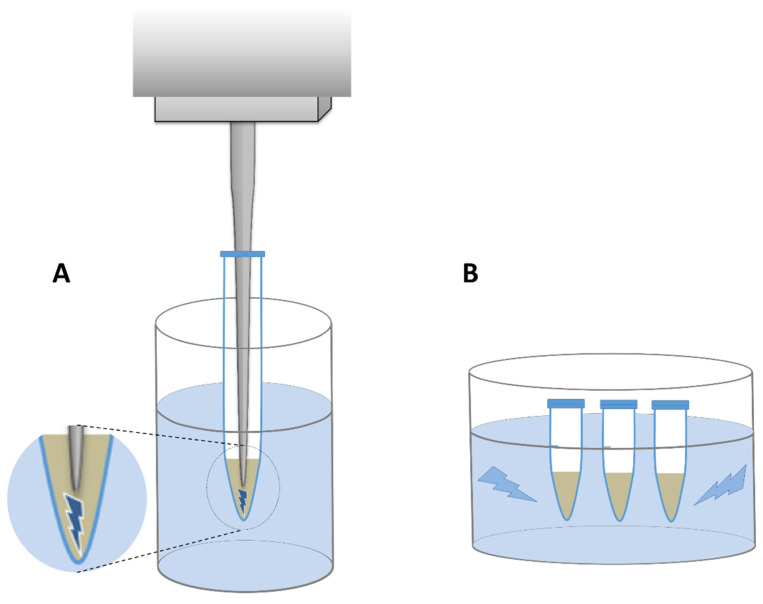
Schematic representation of two sonicator types. (**A**)—probe sonicator, (**B**)—bath sonicator. The area where the ultrasounds generated by probe are released was shown in magnification.

In all lysis variants except one (variant A), samples were centrifuged at 5500×*g* for 10 min before mechanical lysis in order to minimize the physical damage to DNA already present in the supernatant. The suspensions were then withdrawn and collected in separate tubes. The remaining pellets were resuspended with extra 500 µL LS buffer each time and subjected to mechanical lysis with the parameters described in detail in Table 2. Two types of sonicators were used: a probe sonicator with MS 73 probe (Bandelin electronic GmbH & Co. KG, Berlin, Germany) and a bath sonicator Sonic-0,5 (Polsonic, Warsaw, Poland) (Figure 3). In the former, cells were lysed by sonication from 0.5 to 4 min in total depending on the sonication variant (Table 2), with 40% amplitude and without any pause, while the latter one was set with a default power and frequency of 40 kHz, and the sonication lasted for 2 to 6 min in total.

In lysis variant H, zirconia/silica beads (from Bead-Beat Micro AX Gravity kit, A&A Biotechnology) were used, and the lysis was performed in Thermomixer Compact (Eppendorf, Hamburg, Germany) set at 14,000 rpm and 50 °C. After 20 min of shaking, the beads were washed twice with 500 µL LS lysis buffer in order to collect any residual DNA from the beads. In lysis variant 0, only the enzymatic lysis was performed according to manufacturer’s procedure.

For all sonication variants, fractions ‘before’, ‘mid’, and ‘after’ mechanical lysis were combined in a single 5 mL-tube together with 2 µL RNase A (10 mg/mL). Lysates were incubated at room temperature for the entire process of lysis and 10 min after it, and then centrifuged at 12,000×*g* for 5 min. Starting from loading lysates onto columns, the next purification steps were performed according to GMA Bacteria+ protocol. DNA precipitate was resuspended in 50 µL Tris buffer.

Quantification of methanogenic archaea was done according to the same protocol as described previously. Results were calculated into the number of cells in 1 g of droppings.

Quantification of *Salmonella* and the internal control IC2 (*invA*-positive plasmid) was performed using qPCR with the same thermal parameters as described for *mcrA* gene. The reaction mixture was: 15 µL RT HS-PCR Mix Probe (A&A Biotechnology), 0.5 µM of primers Sal_F5b and Sal_R5b, 0.1 µM of Sal_probe1 (Table 1), 1 µL of DNA, and water to reach a final volume of 30 µL. The standard curve was prepared with the use of serial dilutions of the *invA*-positive plasmid linearized by MssI restriction enzyme (Thermo Fisher Scientific, Waltham, MA, USA). Results were calculated into number of *invA* copies in the final volume of total DNA. A *t*-test for independent samples was used to determine statistically significant differences following application of each mechanical lysis method.

### 2.4. Additional Elution of DNA

A total of 10 fresh samples of droppings, from L1 to L10, were collected from the floor of the same henhouse of free-range chickens as samples B1–16 and E1–3. DNA extraction for each dropping sample was performed according to the workflow described in the previous experiment for lysis variants 0 and E, with the only difference being that instead of 200 mg only 100 mg was weighed out. All samples in lysis variant E were subjected to an additional elution step by pouring extra 1 mL of K3 elution buffer once more onto purification columns. Second eluates collected in separate tubes followed the same precipitation process as did the first ones. Absolute quantification of methanogenic archaea was performed in the following days in order to avoid the potential negative effect of storage. Results were calculated into the number of cells in 1 g of droppings. Results for both eluates in variant E were added to each other. Statistical analysis was performed by using a *t*-test for independent samples.

## 3. Results

### 3.1. Sample Screening

Median of measurements of DNA yield of samples B1-16 isolated with the Easy-DNA kit, and with the Easy-DNA kit together with Anti-Inhibitor were 309.84 and 90.86 ng/µL, respectively. The median of A260/280 ratios were 1.24 and 1.50, and the medians of A260/230 ratios were 0.55 and 0.64, respectively (Table S1). Values of DNA yield after isolation with the Easy-DNA kit varied significantly between the samples, and even between the technical replicates. DNA purity was mostly low, and the UV spectra from Nanodrop spectrophotometer strongly indicated DNA contamination with polyphenolic compounds known as potential PCR inhibitors. It is worth mentioning that some DNA samples were visually brown.

After additional purification with the Anti-Inhibitor kit, all DNA samples became colorless, and the values of purity improved, although yield measurements dropped significantly. This suggests that the initial yield measurements were overstated by the contaminants. Polyphenolic compounds could potentially influence OD_600_ and overestimate a nucleic acid concentration.

**Figure 4:**
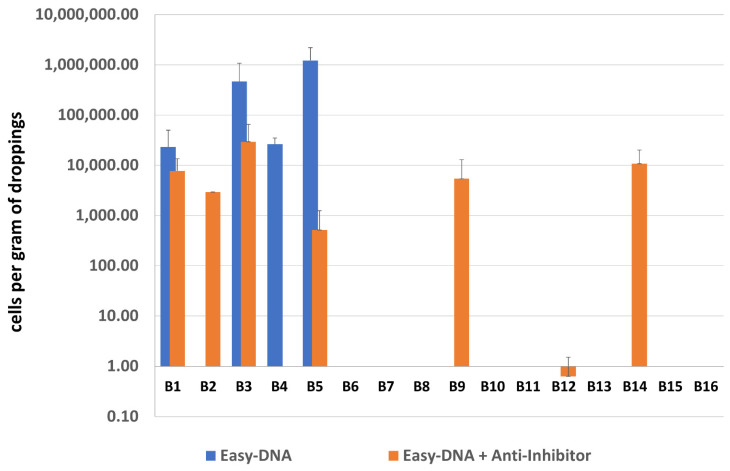
The quantification results of methanogenic archaea in samples B1–B16.

Results of sample screening by qPCR for both types of DNA purification are presented in Figure 4. Eight out of 16 samples after both single and double purification were negative for methanogenic archaea. The occurrence of archaea in sample B12 was questionable, as indicated only by an uncertain result obtained using an additionally purified DNA template. The remaining 7 samples gave positive results, including three samples positive only after using the Anti-Inhibitor purification kit, however it is difficult to conclude with high certainty as to which procedure—with single or double purification—is better since e.g., samples B2 and B4 gave opposing results.

### 3.2. DNA Purification Efficiency Testing

The quantity and quality of DNA obtained in exp. no. 1 were not satisfactory. Therefore, two other commercially available and less expensive kits that were also never tested in similar studies were here evaluated as a potentially better alternative. The B7 sample, which had previously been confirmed negative for the presence of methanogenic archaea, was used for further experiments and inoculated with a known amount of *mcrA*-positive plasmid. The purpose of this study was to assess the efficiency of purification of DNA isolated using selected kits, as well as the real-time PCR performance in detecting of methanogens in feces-derived DNA templates.

**Figure 5:**
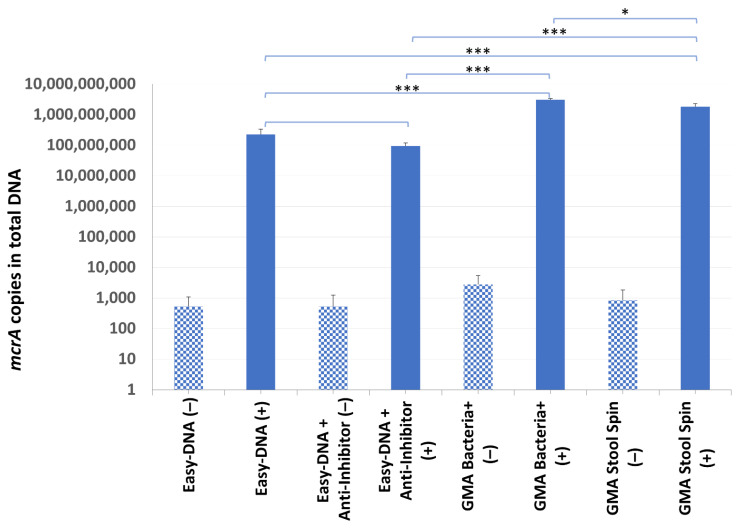
The *mcrA* quantification results in sample B7. Statistical significance between samples with IC (+) isolated with the use of different kits is marked by asterisks. Patterned bars (−) represent archaeal DNA released as a result of a single freeze–thaw cycle. Values of * *p* < 0.05, and *** *p* < 0.001 were regarded as significant. NS; not-significant.

DNA from sample B7 was successfully isolated by all kits, although its yield and purity varied significantly (Table 3). The *mcrA* gene was found in all of the samples, including the non-inoculated ones (from 9.51 × 10^3^ to 5.07 × 10^4^ cells per gram of droppings). It was probably due to freezing and thawing of the B7 droppings, which led to a release of DNA from archaea present in the sample but undetected in the screening experiment. Although the total DNA yield was lower after additional purification of the Easy-DNA template with the Anti-Inhibitor kit (which may have been associated with the removal of some contaminants), no significant differences between the purification efficiency test results were observed (2.26 × 10^8^ copies of *mcrA* gene compared to 9.48 × 10^7^ copies of *mcrA* gene in samples with additional purification). Even though the highest total DNA yield was obtained with the Easy-DNA kit, the quantification results of recovered *mcrA*-positive plasmid gave higher values for the other two kits: GMA Stool Spin recovered 8 times more, and GMA Bacteria+ recovered 14 times more *mcrA* gene copies than the Easy-DNA kit (Figure 5).

Apart from giving the best IC recovery results, the GMA Bacteria+ kit was chosen for further experiments due to column size, which was bigger, and—in theory—could hold more DNA than columns of the GMA Stool Spin kit. In addition, its size allowed for pouring the entire pooled supernatant (approx. 2 mL) onto the column at once, and application of gravity flow instead of centrifuging minimized the necessary manual work. Moreover, elution of DNA in a large volume and its further precipitation allows us to be flexible in deciding of the final volume and concentration of template DNA. On the other hand, despite the manufacturer’s assurance that the GMA Bacteria+ kit columns can hold up to 20 µg of DNA, a total DNA yield was 2.57 µg, which was unsatisfactory, especially considering the much higher phenol-chloroform extraction results. This issue was addressed in the following experiment.

### 3.3. Improvements to DNA Extraction

Due to peculiar molecular composition of the archaeal cell wall, which makes it difficult to disrupt, a mechanical lysis step has been added to the isolation procedure, namely: sonication by different variants or shaking with zirconia/silica beads, both of which were incorporated into the best working DNA isolation kit, i.e., Genomic Mini AX Bacteria+.

**Figure 6:**
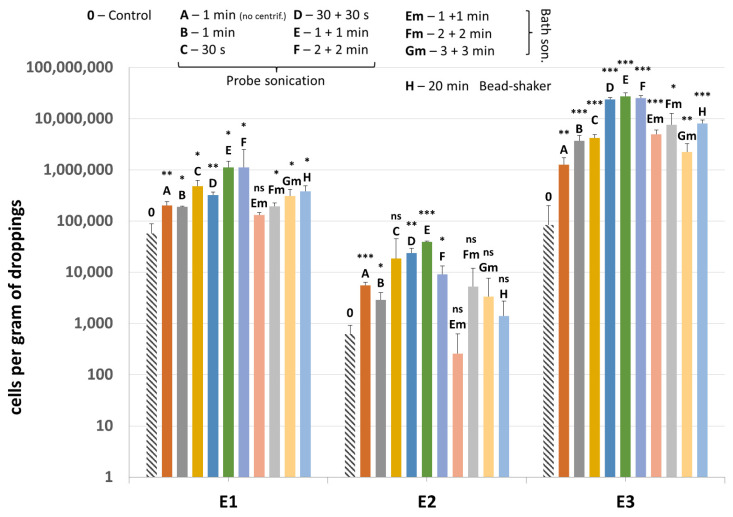
The quantification results of methanogenic archaea in samples E1–E3 subjected to different variants of mechanical lysis. Statistical significance between the non-mechanically-lysed controls (0) and other variants (A–H) is marked by asterisks. Values of * *p* < 0.05, ** *p* < 0.01 and *** *p* < 0.001 were regarded as significant. NS; not-significant.

Methanogenic archaea were detected in all samples (E1–E3) and all lysis variants tested, including the non-mechanically-lysed controls (variants 0). Quantification of the *mcrA* gene revealed that the sample type and cell lysis variant are both substantial (Figure 6). Moreover, sample E3, which consisted mainly of pure dropping content gave much higher results than sample E2, which was rich in undigested plant debris, mostly grass.

Comparing the lysis variants, the highest average amount of methanogenic archaea was detected in the sonication variant E, although variants D and F also gave high values. This means that sonication by probe performed twice for 1 min gave the best results, leading on average to even a 135.4-fold increase in detection of the archaeal DNA compared to control using only enzymatic lysis (Table 4). In lysis variant A of E3 sample, the archaeal DNA was detected in a smaller amount than in variant B, which means that drawing out the lysates prior to mechanical lysis could be an important step, especially since results of variant 0 indicated that the archaeal cell wall (to some extent) disintegrates and releases DNA due to enzymatic lysis only. The ultrasound bath, which is naturally less damaging to microbial cell walls, was not as effective as probe sonication, even if the lysis was extended to 6 min in total. The bead-shaking method gave similar results to bath sonication.

**Figure 7:**
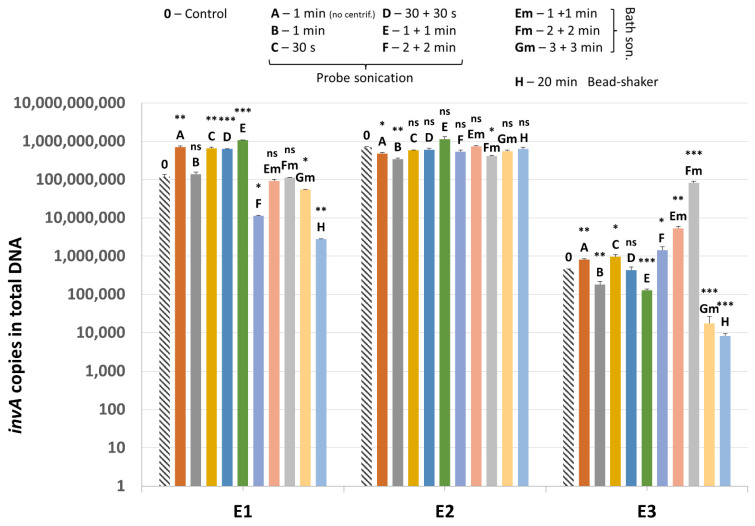
The quantification results of *invA* (IC2) in samples E1–E3. Statistical significance between the non-mechanically-lysed controls (0) and other variants (A–H) is marked by asterisks. Values of * *p* < 0.05, ** *p* < 0.01 and *** *p* < 0.001 were regarded as significant. ns; not-significant.

Results of quantification of *invA* gene present in the plasmid added to samples and used as an additional internal control (IC2) are shown in Figure 7. In sample E2, results are quite homogenous between most variants of mechanical lysis as well as the non-mechanically-lysed control (variant 0).

In sample E1, probe sonication gave generally higher values for *invA* gene detection compared to bath sonication and bead-shaking. Only in lysis variants F and H did the mean value for IC2 drop significantly, probably as a result of plasmid degradation. As for the E3 sample, bath sonication resulted in higher recovery of the *invA*-positive plasmid overall. Samples without the *invA*-positive plasmid tested negative for the presence of *Salmonella* sp.

### 3.4. Additional Elution of DNA

**Figure 8:**
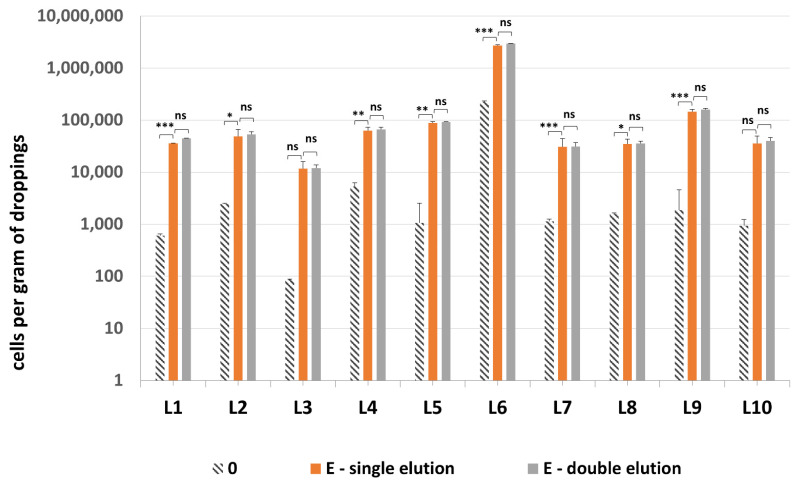
The quantification results of methanogenic archaea in samples L1–L10. Statistical significance between the non-mechanically-lysed controls (0), lysis variants E with single and double elution is marked by asterisks. Values of * *p* < 0.05, ** *p* < 0.01 and *** *p* < 0.001 were regarded as significant. ns; not-significant.

All 10 samples tested positive for presence of methanogenic archaea, with one sample—droppings L6—being the most densely colonized (Figure 8). Quantification of methanogenic archaea in samples L1–10 confirmed the necessity of an additional sonication step by probe, since the difference between sonicated and non-sonicated samples was even 48.3-fold (Table S2). The addition of second elution of DNA from GMA Bacteria+ kit columns did not improve the quantification results significantly—on average there was only a 1.07-fold increase in *mcrA* values obtained for the combined results of both eluates vs. just the single elution.

This concludes the optimization of the DNA isolation protocol. The final proposals are submitted in Box 1, which comprises the most optimal protocol for total DNA isolation, including archaeal DNA extraction, from chicken dropping samples.

Box 1 **The final protocol for total isolation of microbial DNA from fecal samples.**

I Cell lysis

1. Transfer 100 mg of a fresh dropping sample (or 200 mg if the sample is rich in plant debris) into a 15 mL tube and add 350–500 µL BS suspension buffer up to the point when the mix becomes viscous, 30 µL lysozyme (10 mg/mL), and 7 µL mutanolysin (10 U/µL). Incubate at 37 °C for 15 min and then at 50 °C for 25 min.
2. Add 500 µL LS lysis buffer and 35 µL proteinase K (20 mg/mL). Incubate at 50 °C for 60 min. Mix by hand sporadically.
3. Centrifuge at 5500×*g* for 10 min at room temperature.
4. Transfer the supernatant (1st fraction) into a 2 mL tube, add 5 µL RNase A (10 mg/mL), and leave at room temperature. Add 350 µL LS buffer to the remaining pellet and resuspend it with pipette.
5. Sonicate the resuspended pellet for 1 min with constant pulse at 40% amplitude in a probe sonicator.
6. Centrifuge at 5500×*g* for 10 min at room temperature.
7. Transfer the supernatant (2nd fraction) into 2 mL tube and mix with the 1st fraction. Add 300 µL LS buffer to the remaining pellet and resuspend it with pipette.
8. Repeat steps 5–6.
9. Transfer the supernatant (3rd fraction) into 2 mL tube and mix with the other two fractions. Leave at room temperature. for 10 min.
10. Centrifuge at 12,000×*g* for 5 min at room temperature.

II DNA purification

1. Prepare the purification column by adding 800 µL K1 balancing buffer.
2. Transfer total supernatant onto purification column. Wait until it flows through the column.
3. Add 1.5 mL K2 washing buffer.
4. Repeat washing with 1.5 mL K2 washing buffer.
5. Add 250 µL K3 elution buffer (this will allow one to discard the dead volume of buffer from the column).
6. Place the column into the precipitation tube.
7. Add 1 mL K3 buffer. DNA that flows out of the column becomes the 1st eluate. Close the tube.

III DNA double elution (optional)

1. Transfer the column into another precipitation tube.
2. Add 1 mL K3 buffer (this will result in elution of any residual DNA into the 2nd eluate).

IV precipitation

1. Add 800 µL PM precipitation buffer to each of the eluates.
2. Centrifuge at 10,000×*g* for 10 min.
3. Discard the supernatant.
4. Wash the pellets with 500 µL 70% ethanol.
5. Centrifuge at 10,000×*g* for 3 min.
6. Discard the supernatant.
7. Air dry the pellets for 5 min.
8. Suspend the pellets from 1st and 2nd elution with 50 µL Tris buffer (10 mM Tris HCl, pH 8.5).
9. Store the DNA solutions at −20 °C.

The necessary buffers all originate from Genomic Mini AX Bacteria+ kit (A&A Biotechnology). This study was not sponsored by A&A Biotechnology.

## 4. Discussion

Extraction of good quality DNA with high efficiency is a key factor in any quantitative analysis of microbial communities. However, DNA extraction procedures do not focus the attention they deserve, especially the cell lysis step, which is necessary for effective DNA isolation. Development of the best working DNA isolation method should always come down to finding a good balance between getting the highest DNA yield and its best purity. This study illustrates it perfectly.

### 4.1. Cell Lysis

In case of low abundant microorganisms, like archaea, the problem is not only about recovering as much DNA as possible but also about being able to extract DNA from all kinds of microorganisms at once, both the susceptible and the resistant to lysis. After careful revision we have found that the number of publications in which archaea are present in chicken ceca [3,9,10,11] almost matches the number of those in which archaea are not detected or are detected only in isolated cases/individuals [12,13]. Importantly, the tendency to detect these microorganisms has increased in recent years, which may be related to the increasing use of the bead-beating extraction method. This study shows that once we developed an optimal lysis method, archaea were present in all of the dropping samples. Bear in mind that since ceca create by nature more favorable conditions for archaea to live in than the other parts of intestines—where the intestinal content movement is much quicker, and fermentation is not as pronounced—archaea would be far less abundant in droppings than they are in ceca. However, in contrast to other studies, we still managed to detect them in quite a fair amount; therefore, it can be assumed with high probability that in many of the previous studies the insufficient cell lysis was associated with poor detection of this group of microbes.

There are many ways to extract the archaeal DNA. The simplest methods are boiling or the application of freeze–thaw cycles. The boiling method was reported to be as good as repeated bead-beating [14]. As for the freeze–thaw method, we demonstrated that it affects the archaeal cell wall, causing it to rupture and release some DNA, up to 5.07 × 10^4^ per gram of droppings in B7 sample. For this reason, optimization of extraction methods on previously frozen specimens is unfavorable, although due to logistical reasons, it happens quite frequently [14,15]. This amount of DNA, although exceeding most of the values obtained for fresh samples subjected to enzymatic lysis (i.e., lysis variants 0 in exp. no. 3 and 4), was not sufficient and indicated that a single cycle of freeze–thaw alone was ineffective in total cell disruption. This conclusion can also be supported by other research [16]. Thermal methods do not provide reproducible results of DNA isolation, moreover they are difficult to standardize, and can cause substantial DNA degradation, thus requiring subsequent DNA sample purification.

Another method of DNA isolation from archaea uses enzymatic lysis reinforced by chemicals [16]. Enzymes that can be used in studies like this one are only limited to proteinase K because archaea are resistant to lysozyme. However, given that the aim of this study was to develop a universal protocol for total microbial DNA isolation from fecal (and intestinal) samples, and that archaea were used as a determinant of isolation protocol usability in microbiome analyses, we included lysozyme (and mutanolysin) treatment.

As was reported in other studies, simple enzymatic/chemical or thermal treatment, and even the best isolation kits, e.g., probably the most widely used QIAamp DNA Stool Mini kit (Qiagen) are not resulting in satisfactory archaeal DNA yield if not implemented with mechanical cell rupture, like bead-beating [14,15]. We used zirconia/silica beads; however, results were not as good as those obtained for probe sonication. Park et al. [10] used a similar simplified bead-beating method, which we named here the “bead-shaking” method to better distinguish these two according to the equipment used (thermo-shaker vs. bead-beater). They used horizontal vortexing for 10 min, but without enzymatic pretreatment and with further incubation of samples at 95 °C for 6 min., followed by purification with the QIAamp Stool Mini kit. Their method allowed to detect *Methanobacteriales* as one of the 7 most abundant chicken cecal microorganisms at the order level by NGS.

Up to date sonication has been used mainly as a method for extraction of proteins or liposomes [17,18,19]. In DNA-based studies sonication is mostly used for preparation of libraries for shotgun metagenomic analyses of microbial communities [20]. To the best of our knowledge, our study is the first that shows potential use of sonication in DNA isolation from archaea and other microbionts. Other papers describing this method focused on bacteria only [21]. Moreover, we first used enzymatic lysis, and collected lysates prior to sonication to protect already extracted DNA from susceptible microbionts, which is a rare approach in the previously described studies. It needs to be highlighted that the probe sonication procedure required much more attention to detail than other methods. Although it is much more efficient than the bath sonication, there is some risk of contamination between the samples. The probe has to be carefully decontaminated with chemicals degrading DNA, and then washed and dried, which significantly increases the hands-on time of this mechanical lysis procedure.

### 4.2. DNA Purification

High quality DNA is characterized by a lack of contaminants such as proteins and phenols, known as polymerase inhibitors. A study carried out by Scupham et al. [5] indicated that the Easy-DNA kit (Invitrogen) was one of the most effective kits for total DNA isolation from cecal contents in bird gut microbiota analyses. According to authors it was even better than the QIAamp DNA Stool Mini kit (Qiagen), which nowadays seems to be predominantly used in this kind of research [22]. However, the Easy-DNA kit utilizes phenol-chloroform DNA isolation method and, as we rightly suspected, DNA isolated this way required additional purification from phenol residues before qPCR. For this reason, the main focus was thereafter put back on kits in which purification proceeds with adsorptive columns.

The main downside of column-based kits is their susceptibility to overload, which did not remain unnoticed. Our study started off with the recommended 100 mg of droppings in the screening experiment. We then reduced it to 55 mg to avoid column overloading, which could in turn falsely underestimate the DNA purification efficiency rates of column-based kits compared to phenol-chloroform extraction (exp. no. 2). Then, we tested the maximal capacity of columns by taking 200 mg of droppings in exp. no. 3, and we noticed a big difference in DNA isolation efficiency between samples varying in texture and plant debris content. In this way we also addressed the issue of potential influence of different diets and breeding systems. Only in sample E2, with the highest visual amount of plant debris, were the quantification of added vs. recovered *invA*-positive plasmids similar. This suggested that due to a high proportion of undigested grass to actual fecal content, the amount of extracted DNA was more optimal, whereas in samples E1 and E3 the amount of released DNA was higher than the maximum capacity of the GMA Bacteria+ kit columns; thus, some DNA (including the *invA*-positive plasmid) was lost during washing. Therefore, the results from sample E2 were the most meaningful, whereas results from E1 and E3, however agreeable in proportions of detected archaea, may be just an addition to the final conclusion as to which mechanical lysis method works best. For this reason we recommend adding an internal control of isolation process to every survey in order to exclude any detrimental factors like incorrect sample size vs. its content.

Although commercially available column-based extraction kits are effective and increasingly used for DNA isolation, some studies report loss of significant amounts of DNA due to poor elution [15]. However, in our study double elution increased quantification results of methanogens by only 1.07-fold, which suggests that the main loss of DNA was an effect of column overload only.

## 5. Conclusions

DNA of methanogenic archaea was detected (with rising success rate) in all four surveys. This should come as no surprise since all dropping samples were collected from the same chicken coop. However, apart from differences in the prevalence of archaea in individual dropping samples tested (which may be partly due to inter-individual variability), significant differences resulting from the procedure of DNA extraction were also noted. The results of quantification of archaea in non-mechanically-lysed samples were inconsistent. In the non-optimized screening experiment, more than half of the samples was most likely falsely negative, while in the subsequent experiments the number of detected archaea in the samples tested reached log4. After an extra 2-min-log sonication step, the detection values improved even more, rising up to 2.43 × 10^6^ on average in experiments no. 3 and 4 combined. Interestingly, our results from the dropping samples are similar to those reported by Saengkerdsub et al. for chicken ceca, where concentration of archaea were also around 10^5^–10^7^ cells per gram of cecal material [3]. These authors performed qPCR on the gene encoding 16S rRNA, assuming that methanogens usually have one copy of this gene per genome. However, it is now known that archaea usually have 2–4 copies, so the actual number of methanogens detected in the aforementioned study is slightly lower [23]. In addition, of all the samples tested in our study, sample E3, which was most likely cecal droppings, outdid others in the archaeal cell counts. Cecal droppings, unlike fecal ones, were shown to reflect cecal microbiota, especially rich in hydrogen consuming anaerobes, which explains high concentration of methanogenic archaea in this particular sample [24].

In summary, the presented study demonstrated the efficiency of using an ultrasonic probe for total DNA extraction from dropping samples. To the best of our knowledge, this is the first study that compares the reliability of the sonication method in combination with commercially available kits. The newly developed extraction method was found to be efficient in isolating high-yield DNA, and moreover, it is able to extract DNA from archaeal cells, which are particularly difficult to lyse and thus could potentially be overlooked or underestimated in quantitative analyses. Considering high qPCR detection of the *mcrA* gene from methanogenic archaea, often exceeding the values reported in previous studies, DNA extracted by using our protocol can be successfully applied for microbiome studies.

## Figures and Tables

**Table 1:** The list of primers used in this study.

Primer/Probe Name	Sequence	Target	Reference
mcrA_F3	CTTGAARMTCACTTCGGTGGWTC	*mcrA* in methanogenic archaea (271 bp)	this study
mcrA_R1 (=mcrA-rev)	CGTTCATBGCGTAGTTVGGRTAGT		[7]
mcrA_probe2	[6FAM]TM[+G]GHT[+T]C[+T]WYGGWTWCGA[BHQ1] *		this study
Sal_F5b	GTCCAGTTTATCGTTATTACCAAAGG	*invA* in *Salmonella* Typhimurium (200 bp)	modified from [8]
Sal_R5b	ATCGCACCGTCAAAGGA		
Sal_probe1	[6FAM]TTC[+T]CTGGA[+T]GGTATGCCCG[BHQ1] *		this study

* [+]—locked nucleic acid (LNA) nucleotide.

**Table 2:** The mechanical lysis workflow.

Lysis Variant		Step 1—Enzymatic Lysis	Step 2—Centrifuging ^1^	Step 3—Mechanical Lysis I	Step 4—Centrifuging ^2^	Step 5—Mechanical Lysis II	Step 6—Centrifuging ^3^	Step 7—Purification
**Probe sonicator**	**A**	yes	no	1 min	yes	-	-	GMA Bacteria+
	**B**	yes	yes	1 min	yes	-	-	
	**C**	yes	yes	30 s	yes	-	-	
	**D**	yes	yes	30 s	yes	30 s	yes	
	**E**	yes	yes	1 min	yes	1 min	yes	
	**F**	yes	yes	2 min	yes	2 min	yes	
**Bath sonicator**	**Em**	yes	yes	1 min	yes	1 min	yes	
	**Fm**	yes	yes	2 min	yes	2 min	yes	
	**Gm**	yes	yes	3 min	yes	3 min	yes	
**Shaker—H**		yes	yes	20 min, constant			yes, twice	
**Control—0**		yes	yes	-	-	-	-	

^1^—collection of fraction ‘before’; ^2^—collection of fraction ‘mid’; ^3^—collection of fraction ‘after’.

**Table 3:** The results of DNA isolation for sample B7.

DNA Isolation Kit ^1^	Average DNA Concentration [ng/µL]	Total DNA Yield [µg]	A260/280 Ratio
Easy-DNA (−)	483.76 ± 39.244	48.38	1.45
Easy-DNA (+)	811.99 ± 68.165	81.20	1.49
Easy-DNA + Anti-Inhibitor (−)	123.85 ± 0.141	12.38	1.7
Easy-DNA + Anti-Inhibitor (+)	288.63 ± 0.424	28.86	1.78
GMA Bacteria+ (−)	51.44 ± 0.212	2.57	1.85
GMA Bacteria+ (+)	51.42 ± 0.580	2.57	1.85
GMA Stool Spin (−)	7.9 ± 0.212	0.79	1.66
GMA Stool Spin (+)	9.68 ± 0.269	0.97	1.59

^1^ (+)—samples inoculated with 1.30 × 10^9^ *mcrA*-positive plasmid (IC); (−)—samples without the plasmid.

**Table 4:** Fold increase in number of methanogenic archaea in all lysis variants compared to the non-mechanically-lysed controls.

Sample	A	B	C	D	E	F	Em	Fm	Gm	H
**E1**	3.5	3.3	8.3	5.6	19.5	19.5	2.3	3.3	5.4	6.7
**E2**	8.9	4.6	29.7	38.0	62.9	14.5	0.4	8.4	5.3	2.2
**E3**	15.1	44.0	50.1	283.0	323.9	299.2	58.4	90.3	26.5	95.3
**average**	9.1	17.3	29.4	108.9	135.4	111.1	20.4	34.0	12.4	34.7

## Data Availability

Data is contained within this article and Supplementary Materials.

## Supplementary Materials

The following supporting information can be downloaded at: https://www.mdpi.com/article/10.3390/microorganisms10030523/s1, Table S1: DNA yield and purity of samples B1-16. Table S2: Fold increase in number of methanogenic archaea in lysis variant E with single and double elution, compared to the non-mechanically lysed controls.
